# 4-[(*E*)-(4-Hy­droxy-2-oxo-2*H*-chromen-3-yl)methyl­idene­amino]-1,5-dimethyl-2-phenyl-1*H*-pyrazol-3(2*H*)-one monohydrate

**DOI:** 10.1107/S160053681003480X

**Published:** 2010-09-04

**Authors:** Mohammad Asad, Chuan-Wei Oo, Hasnah Osman, Ching Kheng Quah, Hoong-Kun Fun

**Affiliations:** a School of Chemical Sciences, Universiti Sains Malaysia, 11800 USM, Penang, Malaysia; b X-ray Crystallography Unit, School of Physics, Universiti Sains Malaysia, 11800 USM, Penang, Malaysia

## Abstract

In the title compound, C_21_H_17_N_3_O_4_·H_2_O, the coumarin ring system is almost planar (r.m.s. deviation = 0.002 Å) and makes dihedral angles of 1.50 (7) and 57.75 (7)° with the pyrazole and phenyl rings, respectively. The dihedral angle between the pyrazole and phenyl rings is 56.60 (9)°. The pyrazole ring adopts a twisted comformation. The mol­ecular conformation is stabilized by intra­molecular N—H⋯O and C—H⋯O hydrogen bonds, both of which form *S*(6) ring motifs. In the crystal, each water mol­ecule is linked to its adjacent organic mol­ecule *via* pairs of O—H⋯O hydrogen bonds. The packing is further consolidated by pairs of inter­molecular C—H⋯O hydrogen bonds, which link the mol­ecules into dimers; the dimers are stacked along the *b* axis.

## Related literature

For general background and biological activity of pyran­ocoumarin and substituted coumarin derivatives, see: Aries (1974 ▶); da Silva *et al.* (2009 ▶); Huang *et al.* (2010 ▶); Skulnick *et al.* (1997 ▶); Spino *et al.* (1998 ▶); Kokil *et al.* (2010 ▶); Abdelhafez *et al.* (2010 ▶); Honmantgad *et al.* (1985 ▶); Delporte *et al.* (1998 ▶); Ibrahim *et al.* (2006 ▶); Bissonnette *et al.* (2009 ▶). For a related structure, see: Arshad *et al.* (2010 ▶). For reference bond lengths, see: Allen *et al.* (1987 ▶). For the stability of the temperature controller used in the data collection, see: Cosier & Glazer (1986 ▶). For hydrogen-bond motifs, see: Bernstein *et al.* (1995 ▶). For ring conformations, see: Cremer & Pople (1975 ▶).
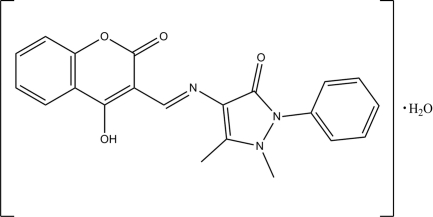


## Experimental

### 

#### Crystal data

C_21_H_17_N_3_O_4_·H_2_O
*M* *_r_* = 393.39
Monoclinic, 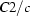

*a* = 35.225 (4) Å
*b* = 6.4269 (7) Å
*c* = 17.6163 (18) Å
β = 108.008 (3)°
*V* = 3792.7 (7) Å^3^
*Z* = 8
Mo *K*α radiation
μ = 0.10 mm^−1^
*T* = 100 K
0.19 × 0.13 × 0.12 mm

#### Data collection

Bruker SMART APEXII CCD area-detector diffractometer
Absorption correction: multi-scan (*SADABS*; Bruker, 2009 ▶) *T*_min_ = 0.981, *T*_max_ = 0.989
35398 measured reflections
5044 independent reflections
3412 reflections with *I* > 2σ(*I*)
*R*_int_ = 0.052

#### Refinement

*R*[*F* ^2^ > 2σ(*F* ^2^)] = 0.051
*wR*(*F* ^2^) = 0.172
*S* = 1.03
5044 reflections
268 parameters
H atoms treated by a mixture of independent and constrained refinement
Δρ_max_ = 0.40 e Å^−3^
Δρ_min_ = −0.66 e Å^−3^

### 

Data collection: *APEX2* (Bruker, 2009 ▶); cell refinement: *SAINT* (Bruker, 2009 ▶); data reduction: *SAINT*; program(s) used to solve structure: *SHELXTL* (Sheldrick, 2008 ▶); program(s) used to refine structure: *SHELXTL*; molecular graphics: *SHELXTL*; software used to prepare material for publication: *SHELXTL* and *PLATON* (Spek, 2009 ▶).

## Supplementary Material

Crystal structure: contains datablocks global, I. DOI: 10.1107/S160053681003480X/hb5618sup1.cif

Structure factors: contains datablocks I. DOI: 10.1107/S160053681003480X/hb5618Isup2.hkl

Additional supplementary materials:  crystallographic information; 3D view; checkCIF report

## Figures and Tables

**Table 1:** Hydrogen-bond geometry (Å, °)

*D*—H⋯*A*	*D*—H	H⋯*A*	*D*⋯*A*	*D*—H⋯*A*
O1*W*—H1*OW*⋯O4	0.87	2.06	2.923 (2)	173
O1*W*—H2*OW*⋯O2	0.88	2.03	2.899 (2)	170
N1—H1*N*1⋯O3	0.93 (3)	1.79 (3)	2.6132 (18)	146 (2)
C3—H3*A*⋯O2^i^	0.93	2.60	3.450 (2)	153
C10—H10*A*⋯O4	0.93	2.35	3.007 (2)	127

Symmetry code: (i) 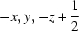.

**Fig. 1.**
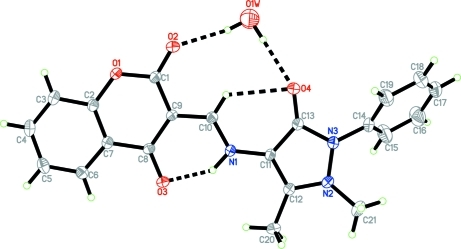
The molecular structure of the title compound showing 50% probability displacement ellipsoids for non-H atoms and the atom-numbering scheme. Hydrogen bonds are shown as dashed lines.

**Fig. 2.**
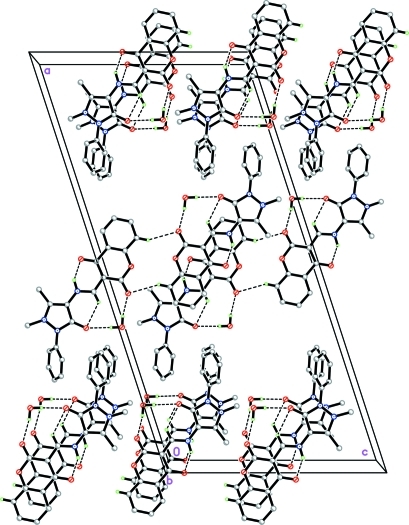
The crystal structure of the title compound viewed along the b axis. H atoms not involved in hydrogen bonding (dashed lines) have been omitted for clarity.

## supplementary crystallographic information

### Comment

A number of pyranocoumarin and substituted coumarin derivatives reported to possess multiple biological activities (Aries, 1974) are used in the treatment of vitiligo (da Silva *et al.*, 2009) and other dermal diseases. Coumarins show various activities such as anticancer (Huang *et al.*, 2010), anti-HIV agents (Skulnick *et al.*, 1997; Spino *et al.*, 1998), antifungal (Kokil *et al.*, 2010), anticoagulant (Abdelhafez *et al.*, 2010), antibacterial (Honmantgad *et al.*, 1985), antipyretic (Delporte *et al.*, 1998), analgesic (Ibrahim *et al.*, 2006) and anti-inflammatory (Bissonnette *et al.*, 2009) properties.

The title compound (Fig. 1) consists of a 4-[(*E*)-(4-hydroxy-2-oxo-2*H*- chromen-3-yl)methylidene]amino-1,5-dimethyl-2-phenyl-1,2-dihydro-3*H*- pyrazol-3-one molecule and a water molecule in the asymmetric unit. The coumarin ring system (C1—C9/O1/O2) is almost planar with a maximum deviation of 0.003 (1) Å for atom C7 and makes dihedral angles of 1.50 (7) and 57.75 (7)° with least-squares planes of pyrazole (N2/N3/C11—C13) and phenyl (C14—C19) rings, respectively. The dihedral angle between least-squares planes of pyrazole and phenyl rings is 56.60 (9)°. The comformation of pyrazole ring is twisted as reflected by the puckering parameters, Q = 0.0683 (16) Å and Θ = 22.9 (14)° with torsion angle C12–N2–N3–C13 being -8.12 (18) °. The crystal structure is stabilized by intramolecular N1–H1N1···O3 and C10–H10A···O4 hydrogen bonds, forming S(6) ring motifs (Bernstein *et al.*, 1995).

In the solid state (Fig. 2), water molecules are linked to main molecules *via* intermolecular O1W–H1OW···O4 and O1W–H2OW···O2 hydrogen bonds. The crystal packing is further consolidated by pairs of intermolecular C3–H3A···O2 hydrogen bonds linking the molecules into dimers which are stacked down the *b* axis.

### Experimental

3-Formyl-4-hydroxycoumarin (0.52 mmol, 100 mg) was dissolved in methanol (10 ml) and 4-aminoantipyrine (0.52 mmol, 106 mg) was then added to the mixture. The reaction mixture was refluxed on water bath for 2 h. The precipitated yellow solid was filtered and washed with ethanol to afford the product which was recrystallized from chloroform to reveal yellow blocks of (I) in 70% yield.

### Refinement

Atom H1N1 was located from the difference Fourier map and refined freely. Atoms H1OW and H2OW were located from the difference Fourier map and refined using a riding model, with *U*_iso_(H) = 1.5 *U*_eq_(O). The remaining H atoms were positioned geometrically and refined using a riding model, with C—H = 0.93–0.97 Å and *U*_iso_(H) = 1.2 or 1.5 *U*_eq_(C). A rotating-group model was applied for the methyl groups. The highest residual electron density peak is located 0.73 Å from C7 and the deepest hole is 0.59 Å from O1W.

### Crystal data

**Table:**

C_21_H_17_N_3_O_4_·H_2_O	*F*(000) = 1648
*M**_r_* = 393.39	*D*_x_ = 1.378 Mg m^−^^3^
Monoclinic, *C*2/*c*	Mo *K*α radiation, λ = 0.71073 Å
Hall symbol: -C 2yc	Cell parameters from 6036 reflections
*a* = 35.225 (4) Å	θ = 2.4–29.9°
*b* = 6.4269 (7) Å	µ = 0.10 mm^−^^1^
*c* = 17.6163 (18) Å	*T* = 100 K
β = 108.008 (3)°	Block, yellow
*V* = 3792.7 (7) Å^3^	0.19 × 0.13 × 0.12 mm
*Z* = 8	

### Data collection

**Table:**

Bruker SMART APEXII CCD area-detector diffractometer	5044 independent reflections
Radiation source: fine-focus sealed tube	3412 reflections with *I* > 2σ(*I*)
graphite	*R*_int_ = 0.052
φ and ω scans	θ_max_ = 29.0°, θ_min_ = 2.4°
Absorption correction: multi-scan (*SADABS*; Bruker, 2009)	*h* = −47→48
*T*_min_ = 0.981, *T*_max_ = 0.989	*k* = −8→8
35398 measured reflections	*l* = −23→24

### Refinement

**Table:**

Refinement on *F*^2^	Primary atom site location: structure-invariant direct methods
Least-squares matrix: full	Secondary atom site location: difference Fourier map
*R*[*F*^2^ > 2σ(*F*^2^)] = 0.051	Hydrogen site location: inferred from neighbouring sites
*wR*(*F*^2^) = 0.172	H atoms treated by a mixture of independent and constrained refinement
*S* = 1.03	*w* = 1/[σ^2^(*F*_o_^2^) + (0.0911*P*)^2^ + 2.8654*P*] where *P* = (*F*_o_^2^ + 2*F*_c_^2^)/3
5044 reflections	(Δ/σ)_max_ < 0.001
268 parameters	Δρ_max_ = 0.40 e Å^−^^3^
0 restraints	Δρ_min_ = −0.66 e Å^−^^3^

### Special details

**Table:**

Experimental. The crystal was placed in the cold stream of an Oxford Cyrosystems Cobra open-flow nitrogen cryostat (Cosier & Glazer, 1986) operating at 100.0 (1) K.
Geometry. All e.s.d.'s (except the e.s.d. in the dihedral angle between two l.s. planes) are estimated using the full covariance matrix. The cell e.s.d.'s are taken into account individually in the estimation of e.s.d.'s in distances, angles and torsion angles; correlations between e.s.d.'s in cell parameters are only used when they are defined by crystal symmetry. An approximate (isotropic) treatment of cell e.s.d.'s is used for estimating e.s.d.'s involving l.s. planes.
Refinement. Refinement of *F*^2^ against ALL reflections. The weighted *R*-factor *wR* and goodness of fit *S* are based on *F*^2^, conventional *R*-factors *R* are based on *F*, with *F* set to zero for negative *F*^2^. The threshold expression of *F*^2^ > σ(*F*^2^) is used only for calculating *R*-factors(gt) *etc*. and is not relevant to the choice of reflections for refinement. *R*-factors based on *F*^2^ are statistically about twice as large as those based on *F*, and *R*- factors based on ALL data will be even larger.

### Fractional atomic coordinates and isotropic or equivalent isotropic displacement parameters (Å^2^)

**Table:**

	*x*	*y*	*z*	*U*_iso_*/*U*_eq_	
O1	0.00970 (3)	0.25568 (17)	0.35782 (6)	0.0201 (3)	
O2	0.07480 (4)	0.2578 (2)	0.41212 (7)	0.0269 (3)	
O3	−0.00816 (3)	0.24563 (17)	0.57775 (6)	0.0212 (3)	
O4	0.15875 (3)	0.2277 (2)	0.65776 (7)	0.0275 (3)	
N1	0.06937 (4)	0.24627 (19)	0.64206 (7)	0.0160 (3)	
N2	0.13874 (4)	0.2275 (2)	0.83849 (8)	0.0246 (3)	
N3	0.16474 (4)	0.2441 (2)	0.79281 (8)	0.0258 (3)	
C1	0.04266 (5)	0.2552 (2)	0.42474 (9)	0.0183 (3)	
C2	−0.02838 (5)	0.2533 (2)	0.36347 (9)	0.0173 (3)	
C3	−0.05929 (5)	0.2540 (2)	0.29108 (9)	0.0210 (3)	
H3A	−0.0537	0.2560	0.2428	0.025*	
C4	−0.09821 (5)	0.2515 (2)	0.29271 (10)	0.0239 (3)	
H4A	−0.1191	0.2517	0.2450	0.029*	
C5	−0.10675 (5)	0.2486 (3)	0.36506 (10)	0.0245 (3)	
H5A	−0.1331	0.2471	0.3654	0.029*	
C6	−0.07578 (5)	0.2482 (2)	0.43627 (10)	0.0207 (3)	
H6A	−0.0815	0.2462	0.4844	0.025*	
C7	−0.03600 (4)	0.2507 (2)	0.43656 (9)	0.0170 (3)	
C8	−0.00234 (5)	0.2493 (2)	0.51080 (9)	0.0160 (3)	
C9	0.03653 (4)	0.2519 (2)	0.50194 (8)	0.0155 (3)	
C10	0.07103 (5)	0.2515 (2)	0.56840 (9)	0.0170 (3)	
H10A	0.0959	0.2551	0.5602	0.020*	
C11	0.10243 (4)	0.2456 (2)	0.71053 (8)	0.0169 (3)	
C12	0.10082 (5)	0.2418 (2)	0.78734 (9)	0.0188 (3)	
C13	0.14344 (5)	0.2403 (3)	0.71201 (9)	0.0202 (3)	
C14	0.20606 (5)	0.1917 (3)	0.82639 (10)	0.0273 (4)	
C15	0.21734 (6)	0.0144 (4)	0.87239 (12)	0.0404 (5)	
H15A	0.1982	−0.0713	0.8826	0.048*	
C16	0.25772 (6)	−0.0340 (4)	0.90322 (13)	0.0462 (5)	
H16A	0.2657	−0.1526	0.9343	0.055*	
C17	0.28592 (5)	0.0944 (4)	0.88758 (11)	0.0376 (5)	
H17A	0.3129	0.0606	0.9074	0.045*	
C18	0.27429 (6)	0.2720 (4)	0.84276 (12)	0.0389 (5)	
H18A	0.2935	0.3589	0.8334	0.047*	
C19	0.23402 (5)	0.3225 (3)	0.81140 (11)	0.0348 (4)	
H19A	0.2261	0.4422	0.7809	0.042*	
C20	0.06538 (5)	0.2483 (3)	0.81601 (10)	0.0260 (4)	
H20A	0.0660	0.1315	0.8504	0.039*	
H20B	0.0415	0.2427	0.7712	0.039*	
H20C	0.0657	0.3751	0.8450	0.039*	
C21	0.15162 (6)	0.3240 (4)	0.91767 (10)	0.0374 (5)	
H21A	0.1323	0.2962	0.9446	0.056*	
H21B	0.1541	0.4716	0.9122	0.056*	
H21C	0.1770	0.2674	0.9482	0.056*	
O1W	0.15736 (5)	0.1509 (3)	0.49339 (10)	0.0663 (6)	
H1OW	0.1580	0.1624	0.5429	0.099*	
H2OW	0.1317	0.1766	0.4741	0.099*	
H1N1	0.0428 (7)	0.242 (3)	0.6406 (15)	0.042 (7)*	

### Atomic displacement parameters (Å^2^)

**Table:**

	*U*^11^	*U*^22^	*U*^33^	*U*^12^	*U*^13^	*U*^23^
O1	0.0195 (5)	0.0275 (6)	0.0130 (5)	−0.0004 (4)	0.0046 (4)	0.0006 (4)
O2	0.0213 (6)	0.0427 (8)	0.0191 (5)	−0.0003 (5)	0.0096 (4)	0.0011 (5)
O3	0.0200 (5)	0.0289 (6)	0.0164 (5)	0.0001 (5)	0.0080 (4)	−0.0006 (4)
O4	0.0190 (6)	0.0454 (8)	0.0194 (5)	−0.0011 (5)	0.0078 (4)	0.0002 (5)
N1	0.0158 (6)	0.0177 (6)	0.0136 (6)	−0.0001 (5)	0.0033 (5)	0.0000 (4)
N2	0.0210 (7)	0.0384 (9)	0.0141 (6)	0.0003 (6)	0.0051 (5)	−0.0001 (5)
N3	0.0172 (6)	0.0425 (9)	0.0164 (6)	0.0005 (6)	0.0033 (5)	0.0015 (6)
C1	0.0197 (7)	0.0196 (7)	0.0153 (6)	−0.0004 (6)	0.0049 (5)	−0.0002 (5)
C2	0.0186 (7)	0.0155 (7)	0.0171 (7)	0.0002 (6)	0.0043 (5)	0.0008 (5)
C3	0.0241 (8)	0.0187 (7)	0.0170 (7)	−0.0006 (6)	0.0018 (6)	0.0003 (6)
C4	0.0227 (8)	0.0184 (7)	0.0239 (8)	0.0011 (6)	−0.0023 (6)	0.0000 (6)
C5	0.0173 (7)	0.0221 (8)	0.0308 (8)	−0.0003 (6)	0.0025 (6)	−0.0011 (6)
C6	0.0197 (7)	0.0200 (8)	0.0224 (7)	0.0002 (6)	0.0065 (6)	−0.0007 (6)
C7	0.0188 (7)	0.0150 (7)	0.0163 (6)	0.0005 (6)	0.0039 (5)	0.0000 (5)
C8	0.0188 (7)	0.0147 (7)	0.0154 (6)	−0.0001 (5)	0.0065 (5)	−0.0003 (5)
C9	0.0170 (7)	0.0163 (7)	0.0133 (6)	−0.0004 (5)	0.0048 (5)	−0.0002 (5)
C10	0.0180 (7)	0.0166 (7)	0.0166 (6)	−0.0007 (6)	0.0056 (5)	0.0005 (5)
C11	0.0167 (7)	0.0183 (7)	0.0145 (6)	−0.0005 (6)	0.0030 (5)	−0.0001 (5)
C12	0.0190 (7)	0.0210 (7)	0.0158 (6)	0.0002 (6)	0.0046 (5)	0.0002 (5)
C13	0.0178 (7)	0.0259 (8)	0.0158 (7)	−0.0009 (6)	0.0037 (5)	0.0009 (6)
C14	0.0185 (8)	0.0411 (10)	0.0191 (7)	−0.0001 (7)	0.0010 (6)	−0.0022 (7)
C15	0.0260 (9)	0.0465 (12)	0.0448 (11)	−0.0014 (8)	0.0052 (8)	0.0132 (9)
C16	0.0326 (11)	0.0538 (14)	0.0462 (12)	0.0088 (10)	0.0034 (9)	0.0155 (10)
C17	0.0217 (8)	0.0586 (13)	0.0287 (9)	0.0060 (9)	0.0022 (7)	−0.0027 (9)
C18	0.0220 (9)	0.0563 (14)	0.0369 (10)	−0.0061 (8)	0.0071 (8)	−0.0008 (9)
C19	0.0251 (9)	0.0437 (11)	0.0333 (9)	−0.0014 (8)	0.0056 (7)	0.0051 (8)
C20	0.0256 (8)	0.0354 (9)	0.0197 (7)	0.0016 (7)	0.0112 (6)	−0.0004 (7)
C21	0.0329 (10)	0.0572 (13)	0.0189 (8)	−0.0017 (9)	0.0032 (7)	−0.0078 (8)
O1W	0.0463 (10)	0.1070 (17)	0.0457 (9)	0.0156 (10)	0.0145 (8)	−0.0139 (10)

### Geometric parameters (Å, °)

**Table:**

O1—C1	1.3753 (18)	C8—C9	1.425 (2)
O1—C2	1.3755 (19)	C9—C10	1.403 (2)
O2—C1	1.219 (2)	C10—H10A	0.9300
O3—C8	1.2583 (17)	C11—C12	1.3718 (19)
O4—C13	1.2367 (19)	C11—C13	1.437 (2)
N1—C10	1.3175 (19)	C12—C20	1.485 (2)
N1—C11	1.3937 (18)	C14—C19	1.381 (3)
N1—H1N1	0.93 (2)	C14—C15	1.383 (3)
N2—C12	1.364 (2)	C15—C16	1.392 (3)
N2—N3	1.3977 (19)	C15—H15A	0.9300
N2—C21	1.465 (2)	C16—C17	1.383 (3)
N3—C13	1.3890 (19)	C16—H16A	0.9300
N3—C14	1.432 (2)	C17—C18	1.376 (3)
C1—C9	1.4416 (19)	C17—H17A	0.9300
C2—C7	1.394 (2)	C18—C19	1.392 (3)
C2—C3	1.398 (2)	C18—H18A	0.9300
C3—C4	1.380 (2)	C19—H19A	0.9300
C3—H3A	0.9300	C20—H20A	0.9600
C4—C5	1.397 (2)	C20—H20B	0.9600
C4—H4A	0.9300	C20—H20C	0.9600
C5—C6	1.385 (2)	C21—H21A	0.9600
C5—H5A	0.9300	C21—H21B	0.9600
C6—C7	1.400 (2)	C21—H21C	0.9600
C6—H6A	0.9300	O1W—H1OW	0.8673
C7—C8	1.469 (2)	O1W—H2OW	0.8763
			
C1—O1—C2	121.45 (12)	C12—C11—N1	125.13 (14)
C10—N1—C11	124.94 (14)	C12—C11—C13	109.24 (13)
C10—N1—H1N1	109.0 (16)	N1—C11—C13	125.59 (13)
C11—N1—H1N1	126.1 (16)	N2—C12—C11	108.80 (14)
C12—N2—N3	107.25 (12)	N2—C12—C20	122.12 (14)
C12—N2—C21	123.53 (15)	C11—C12—C20	129.07 (14)
N3—N2—C21	116.80 (14)	O4—C13—N3	124.51 (15)
C13—N3—N2	110.25 (13)	O4—C13—C11	131.58 (14)
C13—N3—C14	125.28 (14)	N3—C13—C11	103.87 (13)
N2—N3—C14	120.50 (13)	C19—C14—C15	121.39 (17)
O2—C1—O1	115.41 (13)	C19—C14—N3	118.14 (17)
O2—C1—C9	126.19 (14)	C15—C14—N3	120.47 (17)
O1—C1—C9	118.40 (13)	C14—C15—C16	119.12 (19)
O1—C2—C7	122.50 (13)	C14—C15—H15A	120.4
O1—C2—C3	115.85 (14)	C16—C15—H15A	120.4
C7—C2—C3	121.64 (15)	C17—C16—C15	119.9 (2)
C4—C3—C2	118.66 (15)	C17—C16—H16A	120.0
C4—C3—H3A	120.7	C15—C16—H16A	120.0
C2—C3—H3A	120.7	C18—C17—C16	120.30 (18)
C3—C4—C5	120.96 (14)	C18—C17—H17A	119.9
C3—C4—H4A	119.5	C16—C17—H17A	119.9
C5—C4—H4A	119.5	C17—C18—C19	120.48 (19)
C6—C5—C4	119.67 (15)	C17—C18—H18A	119.8
C6—C5—H5A	120.2	C19—C18—H18A	119.8
C4—C5—H5A	120.2	C14—C19—C18	118.77 (19)
C5—C6—C7	120.71 (15)	C14—C19—H19A	120.6
C5—C6—H6A	119.6	C18—C19—H19A	120.6
C7—C6—H6A	119.6	C12—C20—H20A	109.5
C2—C7—C6	118.36 (14)	C12—C20—H20B	109.5
C2—C7—C8	119.30 (14)	H20A—C20—H20B	109.5
C6—C7—C8	122.34 (14)	C12—C20—H20C	109.5
O3—C8—C9	122.90 (14)	H20A—C20—H20C	109.5
O3—C8—C7	120.93 (14)	H20B—C20—H20C	109.5
C9—C8—C7	116.16 (13)	N2—C21—H21A	109.5
C10—C9—C8	121.49 (13)	N2—C21—H21B	109.5
C10—C9—C1	116.33 (13)	H21A—C21—H21B	109.5
C8—C9—C1	122.18 (13)	N2—C21—H21C	109.5
N1—C10—C9	122.09 (14)	H21A—C21—H21C	109.5
N1—C10—H10A	119.0	H21B—C21—H21C	109.5
C9—C10—H10A	119.0	H1OW—O1W—H2OW	94.6
			
C12—N2—N3—C13	−8.12 (18)	C8—C9—C10—N1	0.8 (2)
C21—N2—N3—C13	−151.71 (16)	C1—C9—C10—N1	−179.23 (14)
C12—N2—N3—C14	−166.83 (15)	C10—N1—C11—C12	179.45 (15)
C21—N2—N3—C14	49.6 (2)	C10—N1—C11—C13	−3.2 (2)
C2—O1—C1—O2	−179.86 (13)	N3—N2—C12—C11	6.06 (18)
C2—O1—C1—C9	0.1 (2)	C21—N2—C12—C11	146.60 (17)
C1—O1—C2—C7	0.1 (2)	N3—N2—C12—C20	−174.70 (15)
C1—O1—C2—C3	−179.95 (13)	C21—N2—C12—C20	−34.2 (3)
O1—C2—C3—C4	179.93 (13)	N1—C11—C12—N2	175.77 (14)
C7—C2—C3—C4	−0.1 (2)	C13—C11—C12—N2	−1.98 (18)
C2—C3—C4—C5	0.1 (2)	N1—C11—C12—C20	−3.4 (3)
C3—C4—C5—C6	0.0 (2)	C13—C11—C12—C20	178.85 (16)
C4—C5—C6—C7	0.1 (2)	N2—N3—C13—O4	−171.06 (16)
O1—C2—C7—C6	−179.92 (13)	C14—N3—C13—O4	−13.6 (3)
C3—C2—C7—C6	0.1 (2)	N2—N3—C13—C11	6.66 (17)
O1—C2—C7—C8	−0.3 (2)	C14—N3—C13—C11	164.13 (16)
C3—C2—C7—C8	179.73 (14)	C12—C11—C13—O4	174.61 (18)
C5—C6—C7—C2	−0.1 (2)	N1—C11—C13—O4	−3.1 (3)
C5—C6—C7—C8	−179.68 (15)	C12—C11—C13—N3	−2.89 (17)
C2—C7—C8—O3	−179.56 (14)	N1—C11—C13—N3	179.38 (14)
C6—C7—C8—O3	0.0 (2)	C13—N3—C14—C19	68.3 (2)
C2—C7—C8—C9	0.3 (2)	N2—N3—C14—C19	−136.40 (18)
C6—C7—C8—C9	179.91 (14)	C13—N3—C14—C15	−111.6 (2)
O3—C8—C9—C10	−0.3 (2)	N2—N3—C14—C15	43.7 (2)
C7—C8—C9—C10	179.79 (13)	C19—C14—C15—C16	−0.9 (3)
O3—C8—C9—C1	179.74 (14)	N3—C14—C15—C16	179.04 (19)
C7—C8—C9—C1	−0.2 (2)	C14—C15—C16—C17	−0.1 (3)
O2—C1—C9—C10	−0.1 (2)	C15—C16—C17—C18	1.1 (3)
O1—C1—C9—C10	180.00 (13)	C16—C17—C18—C19	−1.2 (3)
O2—C1—C9—C8	179.89 (15)	C15—C14—C19—C18	0.8 (3)
O1—C1—C9—C8	0.0 (2)	N3—C14—C19—C18	−179.13 (17)
C11—N1—C10—C9	180.00 (14)	C17—C18—C19—C14	0.3 (3)

### Hydrogen-bond geometry (Å, °)

**Table:**

*D*—H···*A*	*D*—H	H···*A*	*D*···*A*	*D*—H···*A*
O1W—H1OW···O4	0.87	2.06	2.923 (2)	173
O1W—H2OW···O2	0.88	2.03	2.899 (2)	170
N1—H1N1···O3	0.93 (3)	1.79 (3)	2.6132 (18)	146 (2)
C3—H3A···O2^i^	0.93	2.60	3.450 (2)	153
C10—H10A···O4	0.93	2.35	3.007 (2)	127

Symmetry codes: (i) −*x*, *y*, −*z*+1/2.
